# Rhombencephalitis and Myeloradiculitis Caused by a European Subtype of Tick-Borne Encephalitis Virus 

**DOI:** 10.3201/eid2512.191017

**Published:** 2019-12

**Authors:** Lorna Neill, Anna M. Checkley, Laura A. Benjamin, M. Trent Herdman, Daniel P. Carter, Steven T. Pullan, Emma Aarons, Katie Griffiths, Bernadette Monaghan, Kushan Karunaratne, Olga Ciccarelli, Jennifer Spillane, David A.J. Moore, Dimitri M. Kullmann

**Affiliations:** University College London Hospitals, London, UK (L. Neill, A.M. Checkley, L.A. Benjamin, M.T. Herdman, B. Monaghan, K. Karunaratne, O. Ciccarelli, J. Spillane, D.A.J. Moore, D.M. Kullmann);; University College London, London (L.A. Benjamin, O. Ciccarelli, J. Spillane, D.M. Kullmann);; Public Health England, Porton Down, UK (M.T. Herdman, D.P. Carter, S.T. Pullan, E. Aarons, K. Griffiths);; London School of Hygiene and Tropical Medicine, London (D.A.J. Moore)

**Keywords:** Encephalitis, Rhombencephalitis, meningitis/encephalitis, Meningoencephalitis, Myeloradiculitis, Tickborne, Phylogeny, Travel-Related Illness, Lithuania, Myelitis, United Kingdom, Vector-borne infections, Viruses

## Abstract

We report a case of a previously healthy man returning to the United Kingdom from Lithuania who developed rhombencephalitis and myeloradiculitis due to tick-borne encephalitis. These findings add to sparse data on tick-borne encephalitis virus phylogeny and associated neurologic syndromes and underscore the importance of vaccinating people traveling to endemic regions.

Tick-borne encephalitis virus (TBEV) is an emerging disease caused by a neurotropic flavivirus; its incidence is increasing in north, central, and eastern Europe (*1**,**2*)*.* Typical resulting neurologic illnesses include meningitis or meningoencephalitis (*3*)*.* Cases peak in the summer, when contact between humans and tick vectors is highest, and infection is associated with time spent in meadows and forests (*1**,**2*)*.* We report a previously healthy 38-year-old man from the United Kingdom who had unusual neurologic manifestations of TBEV after travel to Lithuania. 

The patient, who had received no travel-related vaccinations, traveled to the Kaunas region, where he visited woodlands. He reported having received insect bites on his feet. Seven days after arriving in Lithuania, he developed influenza-like symptoms, which continued after his return to the United Kingdom. Ten days later, he reported neck stiffness, photophobia, slurred speech, tongue deviation to the left, and left leg weakness; the next day, progressive bilateral lower limb weakness in his hips, urinary retention, and constipation developed. At that time, he sought treatment at a hospital. 

On examination, the patient was febrile (38.0°C) and had a peripheral leukocyte count of 15 × 10^9^ cells/L and C-reactive protein of 120 mg/L. Cauda equina syndrome was ruled out by using lumbar-sacral magnetic resonance imaging; results of a computed tomography scan of the head were unremarkable. Pleocytosis was identified in the cerebrospinal fluid (CSF), and the patient was empirically treated with ceftriaxone and acyclovir (Appendix Table). 

Two days after neurologic signs began, the patient became breathless and drowsy. Neurologic examination revealed dysarthria, interrupted saccades, and difficulty with alternating lateral tongue movements. He exhibited a pout reflex and a brisk jaw jerk. Upper limbs had normal tone; power was graded 4+/5 on the Medical Research Council (MRC) scale (https://mrc.ukri.org/research/facilities-and-resources-for-researchers/mrc-scales/mrc-muscle-scale) for shoulder abduction and elbow extension bilaterally but was otherwise normal. The patient had reduced tone in his lower limbs and bilateral proximal muscle weakness affecting hip and knee flexors (MRC grade 1–2/5); distal limb power was less affected (MRC grade 4/5). He was areflexic and had bilateral flexor plantars. Pinprick testing indicated dermatomal sensory loss isolated from L2 to L5 on the left. Forced vital capacity was 800 mL (reference >1,700 mL); therefore, due to respiratory muscle weakness, he was intubated and transferred to a neurology hospital. Repeat CSF testing showed a profile similar to the initial test (Appendix Table)*.* Antituberculosis therapy was added because of the enigmatic etiology. 

Magnetic resonance imaging of the brain and spinal cord demonstrated long-segment myelitis with high T2 signal in the central cord extending from C2 through T12; no intracranial lesions or pathological enhancement were seen (Figure)*.* Neurophysiology test results pointed to a preganglionic lesion, with decreased compound muscle action potentials in the L4–S1 myotomes, in the context of a normal motor conduction velocity and sensory nerve action potential. Mild denervation affecting L4–S1 roots did not explain the patient’s degree of weakness. His neurologic syndrome was consistent with rhombencephalitis and myeloradiculitis. High-dose steroid was added to cover the possibility of neuromyelitis optica. 

**Figure:**
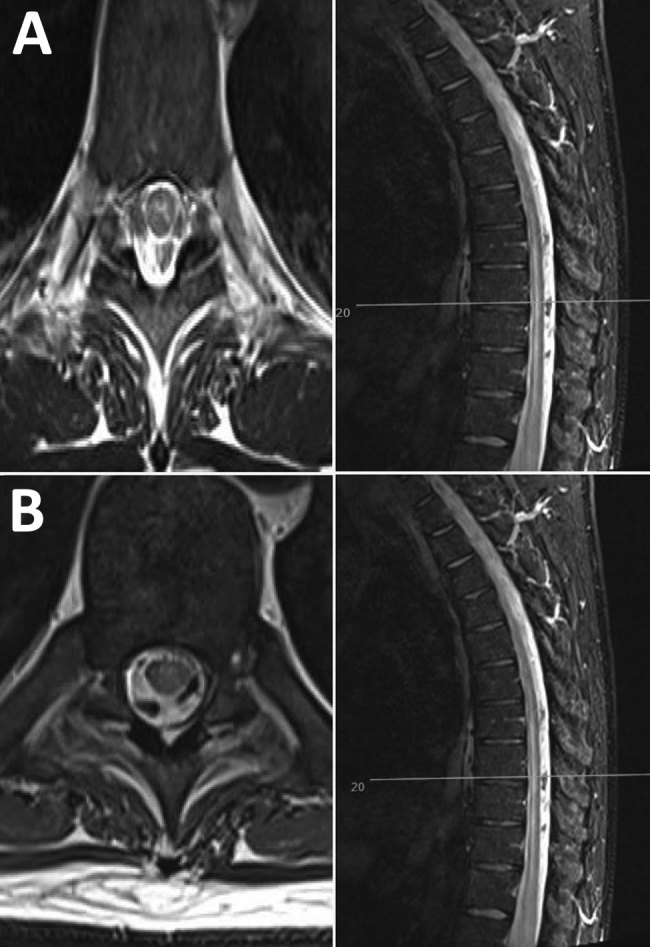
Neurologic manifestations of tick-borne encephalitis in a 38-year-old man from the United Kingdom after travel to Lithuania. A) Magnetic resonance imaging of the brain and spinal cord at onset of neurologic signs, showing possible longitudinal extensive transverse myelitis in the cervical and thoracic cord, with involvement of the central gray matter. B) One month later, increased T2 signal and mild swelling of the central gray matter of the cervical cord have both regressed, with some residual subtle signal changes throughout the spinal cord. Left, axial images; right, sagittal images.

Blood and CSF were screened for inflammatory and infective etiologies (Appendix Table)*.* Serum and urine samples were sent to the Rare and Imported Pathogens Laboratory (Porton Down, UK) for serologic and PCR testing for alphaviruses, flaviviruses, and rickettsial infections. Serum and urine PCR results were positive for TBEV RNA; serum and CSF results were positive for TBEV IgG (Appendix Table). Metagenomic RNA sequencing confirmed TBEV*.* A total of 129 reads (0.01% of total reads) were identified as TBEV, sufficient to elucidate the full envelope gene sequence at a minimum coverage depth of 5× (when mapped to reference sequence GenBank accession no. KC154190.1). No reads were observed for other pathogens. Phylogenetic analysis of the envelope gene revealed the isolate was most closely related to the European TBEV clade (GenBank accession no. MK992869) (Appendix Figure)*.*

Detection of TBEV RNA from both blood and urine is diagnostic of acute TBEV infection (*1*)*.* On day 14, antibiotics, antivirals, and steroids were stopped; antituberculosis therapy had been halted earlier. The patient was extubated on day 17 and has slowly recovered. However, he has residual profound proximal left leg weakness and bladder and bowel dysfunction.

Several subtypes of TBEV cause disease: European, Siberian, and Far Eastern (*1*)*.* Siberian and Far Eastern have been associated with worse outcomes (*1*), but the potentially fatal neurologic complications in this patient are consistent with emerging data indicating that the European subtype causes more severe disease than previously thought (*4**–**6*)*.* In <10% of cases, TBEV targets the anterior horn of the spinal cord, resulting in flaccid poliomyelitis-like paralysis (*3*,*7*), or, rarer still, as in this case, in paralysis of respiratory muscles, requiring artificial ventilation (*3*,*8*,*9*)*.*

Treatment of TBEV is supportive only; vaccination and avoiding mosquito bites are key to disease prevention and control. Although some TBEV-endemic countries have vaccination programs, level of uptake varies (*10*)*.* Public health experts recommend that travelers undertaking high-exposure activities in endemic countries get vaccinated. This case underscores the importance of vaccination among groups of susceptible people and improved awareness of this emerging disease. 

Appendix Details of genomic profile of pathogen and testing results for rhombencephalitis and myeloradiculitis caused by European subtype of tick-borne encephalitis virus.
