# Supplementary material for: Rhombencephalitis and Myeloradiculitis Caused by a European Subtype of Tick-Borne Encephalitis Virus
Source: Emerg Infect Dis. 2019 Dec;25(12):2317–9. doi: 10.3201/eid2512.191017 (PMC6874248; doi:10.3201/eid2512.191017)
Supplement: Appendix — Details of genomic profile of pathogen and testing results for rhombencephalitis and myeloradiculitis caused by European subtype of tick-borne encephalitis virus. [file 19-1017-Techapp-s1.pdf]

Article DOI: https://doi.org/10.3201/eid2512.191017

Rhombencephalitis and Myeloradiculitis Caused by European Subtype of Tick-Borne Encephalitis Virus

Appendix

Appendix Table. Detailed results of laboratory testing*

Results	Blood<br>Day 6†	Urine<br>Day 7†	CSF<br>Day 3†	CSF<br>Day 6†
TBE IgM serology	ND	ND	ND	ND
TBE IgG serology	positive [1 in 3200]	ND	ND	positive [1 in 100]
TBE RNA PCR	positive	positive	negative	negative
Other results	WCC 15×10^9 cells/L<br>Normal limits:<br>Hb, PLT, U&Es, LFTs, bone profile<br>Negative results: NMO/MOG, ANA, ANCA, ACE, onconeuronal antibody, HIV, HTLV-1, syphilis, HHV6, Lyme, rickettsia (spotted fever, typhus), flavivirus (West Nile, sandfly), brucella melitensis		OP: ND<br>WCC: 308×10^6 cells/L (90% monon.)<br>RCC: 12×10^6 cells/L<br>Protein: 1.3g/L<br>Glucose: 2.4mmol/L<br>OCB: ND<br>IgG index: ND	OP: 40+ cm H2O<br>WCC: 430×10^6 cells/L (60% polym.)<br>RCC: 40x10^6/L<br>Protein: 1.0 g/L<br>Glucose: 3.4 (serum: 3.4mmol/L)<br>OCB: no bands<br>IgG Index: 0.66<br>Negative results:<br>Herpes-, entero-, paraechoviruses, TB PCR, CRAG, TB AFB; bacterial and fungal culture

*ACE, angiotensin converting enzyme; AFB, acid fast bacilli; ANA, antinuclear acid; ANCA, antineutrophil cytoplasmic antibody; CRAG, cryptococcal antigen; CRP, C-reactive protein; CSF, cerebrospinal fluid; Hb, haemoglobin; HHV6, Human Herpesvirus 6; HTLV-1, human T-lymphotrophic virus; LFTs, liver function test; MOG, myelin oligodendrocyte glycoprotein antibody; monon., mononuclear cells; ND, not done; NMO, neuromyelitis optica antibody; OCB, oligoclonal bands; OP, opening pressure; PLT, platelet; polym., polymorphonuclear cells; RCC, red cell count; TB, tuberculosis; TBE, Tick-borne encephalitis; U&Es, urea and electrolyte; WCC, white cell count.
†Days after onset of neurologic syndrome.

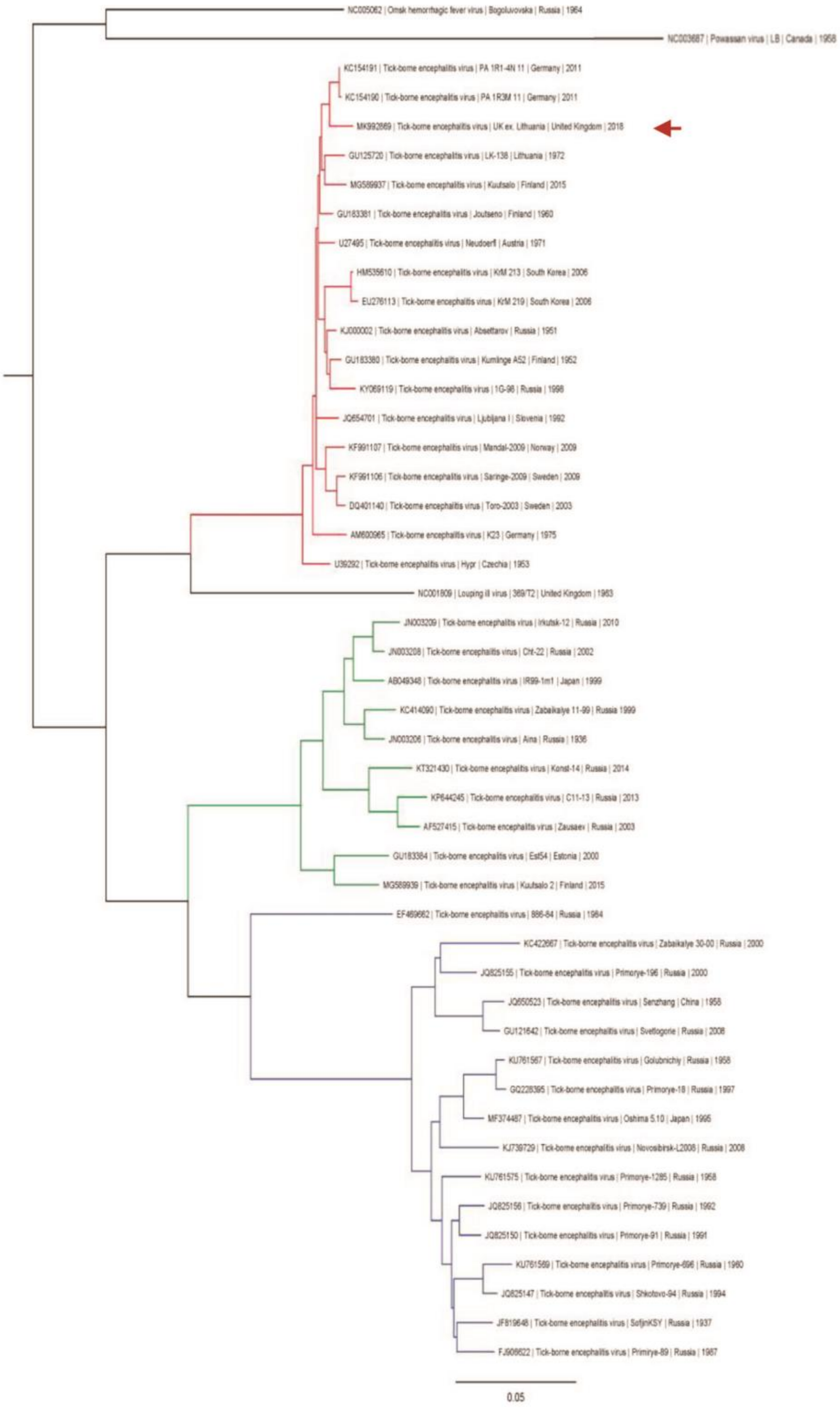

Appendix Figure. Phylogenetic tree of tick-borne encephalitis virus in a 38-year-old man from the United Kingdom after travel to Lithuania. Maximum likelihood phylogenetic tree generated using the GTR (generalized time-reversible) model in MEGA7 (https://www.megasoftware.net) of representative full

envelope genes of TBEV. The European clade is in red, and the isolate of interest is labeled “MK992869” (red arrow). The green nodes are Siberian, and the blue nodes are Far Eastern subtype. All sequences available for GenBank under the accession numbers shown.
